# The Coupling Effects of Surface Plasmon Polaritons and Magnetic Dipole Resonances in Metamaterials

**DOI:** 10.1186/s11671-017-2350-z

**Published:** 2017-11-09

**Authors:** Bo Liu, Chaojun Tang, Jing Chen, Zhendong Yan, Mingwei Zhu, Yongxing Sui, Huang Tang

**Affiliations:** 1 School of Mathematics and Physics, Jiangsu University of Technology, Changzhou, 213001 China; 2 Center for Optics and Optoelectronics Research, Collaborative Innovation Center for Information Technology in Biological and Medical Physics, College of Science, Zhejiang University of Technology, Hangzhou, 310023 China; 3 College of Electronic and Optical Engineering & College of Microelectronics, Nanjing University of Posts and Telecommunications, Nanjing, 210023 China; 4 State Key Laboratory of Millimeter Waves, Southeast University, Nanjing, 210096 China; 5 National Laboratory of Solid State Microstructures and Department of Materials Science and Engineering, Nanjing University, Nanjing, 210093 China

**Keywords:** Metamaterials, Plasmonics, Surface plasmon polaritons, Magnetic dipole resonances, Magnetic field enhancement

## Abstract

We numerically investigate the coupling effects of surface plasmon polaritons (SPPs) and magnetic dipole (MD) resonances in metamaterials, which are composed of an Ag nanodisk array and a SiO_2_ spacer on an Ag substrate. The periodicity of the Ag nanodisk array leads to the excitation of SPPs at the surface of the Ag substrate. The near-field plasmon interactions between individual Ag nanodisks and the Ag substrate form MD resonances. When the excitation wavelengths of SPPs are tuned to approach the position of MD resonances by changing the array period of Ag nanodisks, SPPs and MD resonances are coupled together into two hybridized modes, whose positions can be well predicted by a coupling model of two oscillators. In the strong coupling regime of SPPs and MD resonances, the hybridized modes exhibit an obvious anti-crossing, resulting into an interesting phenomenon of Rabi splitting. Moreover, the magnetic fields under the Ag nanodisks are greatly enhanced, which may find some potential applications, such as magnetic nonlinearity.

## Background

It is well known that naturally occurring materials exhibit the saturation of the magnetic response beyond the THz regime. In light-matter interactions at optical frequencies, the magnetic component of light generally plays a negligible role, because the force exerted by the electric field on a charge is much larger than the force applied by the magnetic field, when light interacts with matter [1]. In the past few years, developing various metallic or dielectric nanostructures with appreciable magnetic response at optical frequencies has been a matter of intense study in the field of metamaterials. Recently, there is increasing interest in optical magnetic field characterization in nanoscale, although it remains a challenge because of the weak matter-optical magnetic field interactions [2]. At the same time, there have also been many efforts to obtain strong magnetic response with magnetic field enhancement in a wide spectrum range from visible [3–22] to infrared [23–44] regime. The physical mechanism underlining strong magnetic response is mainly the excitation of MD resonance in a variety of nanostructures including metal-insulator-metal (MIM) sandwich structures [3, 12, 16, 31, 32, 40], metallic split-ring resonators [29, 30, 36, 41, 42], high-refractive-index dielectric nanoparticles [14, 15, 17, 18, 20, 21], plasmonic nanoantennas [6, 8, 24–26, 28, 34, 37, 43], metamolecules [7, 9, 11, 13, 19, 33, 35, 38], and so on. To obtain strong magnetic response with magnetic field enhancement, MD resonance is also coupled to different narrow-band resonance modes with a high-quality factor, e.g., surface lattice resonances [4, 22, 39, 44], Fabry-Pérot cavity resonances [10, 23], Bloch surface waves [5], and Tamm plasmons [27]. A strong magnetic response with a great enhancement of magnetic fields at optical frequencies will have many potential applications, such as MD spontaneous emission [45–52], magnetic nonlinearity [53–56], optically controlled magnetic-field etching [57], magnetic optical Kerr effect [58], optical tweezers based on magnetic-field gradient [59, 60], circular dichroism (CD) measurement [61], etc. It is well known that plasmonic electric dipole resonance can hugely enhance electric fields in the vicinity of metal nanoparticles, and its coupling to SPPs can further enhance electric fields and generate other interesting physical phenomena. However, there are only a few researches on the coupling effects of SPPs and MD resonances.

In this work, we will numerically demonstrate the huge enhancement of magnetic fields at optical frequencies and the interesting phenomenon of Rabi splitting, due to the coupling effects of SPPs and MD resonances in metamaterials composed of an Ag nanodisk array and a SiO_2_ spacer on an Ag substrate. The near-field plasmon interactions between individual Ag nanodisks and the Ag substrate form MD resonances. The periodicity of the Ag nanodisk array leads to the excitation of SPPs at the surface of the Ag substrate. When the excitation wavelengths of SPPs are tuned to approach the position of MD resonances by changing the array period of Ag nanodisks, SPPs and MD resonances are coupled together into two hybridized modes, whose positions can be well predicted by a coupling model of two oscillators. In the strong coupling regime of SPPs and MD resonances, the hybridized modes exhibit an obvious anti-crossing, resulting into an interesting phenomenon of Rabi splitting. Moreover, the magnetic fields under the Ag nanodisks are greatly enhanced, which may find some potential applications, such as magnetic nonlinearity.

**Fig. 1:**
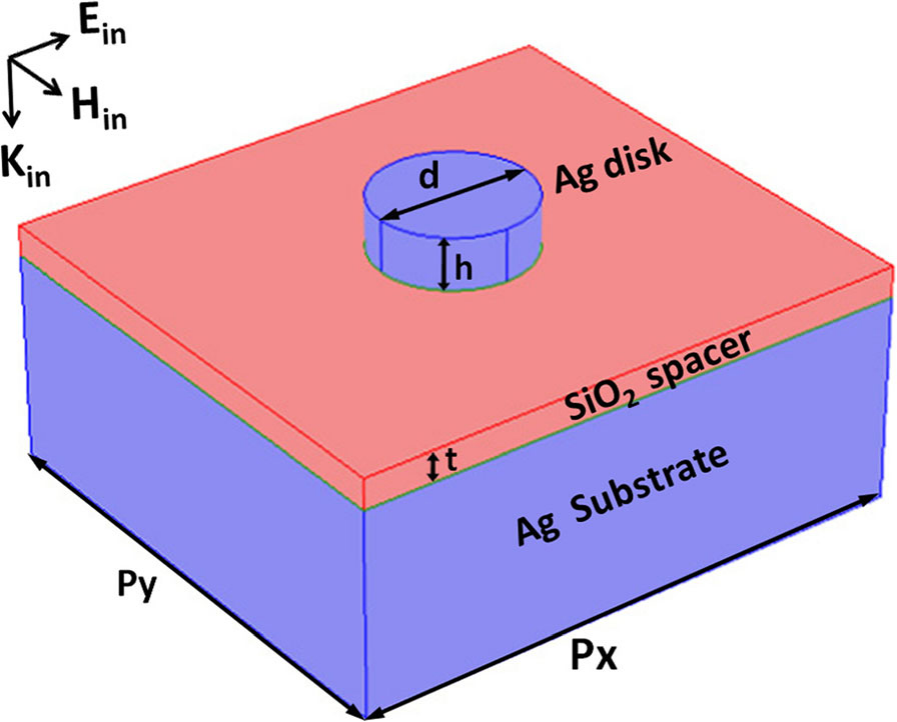
Schematic of metamaterials composed of Ag nanodisks and a SiO_2_ spacer on Ag substrate. Geometrical parameters: *p*_*x*_ and *p*_*y*_ are the array periods along the *x* and *y* directions, respectively; *t* is the thickness of the SiO_2_ spacer; *d* and *h* are the diameter and the height of the Ag nanodisks. ***E***_in_, ***H***_in_, and ***K***_in_ are the electric field, magnetic field, and wave vector of the incident light, which are along the *x*, *y*, and *z* axes, respectively

The unit cell of the designed metamaterials for the coupling effects of SPPs and MD resonances is schematically shown in Fig. 1. The Ag nanodisks lie on the *xy* plane, and the coordinate origin is supposed to be located at the center of the SiO_2_ spacer. The incident light propagates in the negative *z*-axis direction, with its electric and magnetic fields along the *x*-axis and the *y*-axis directions, respectively. The reflection and absorption spectra and the electromagnetic field distributions are calculated by using the commercial software package “EastFDTD,” which is based on finite difference time domain (FDTD) method [62]. In our numerical calculations, the refractive index of SiO_2_ is 1.45, and the frequency-dependent relative permittivity of Ag is taken from experimental data [63]. This work mainly focuses on numerical investigation, but the designed metamaterials should be realized experimentally by the following procedures: the SiO_2_ spacer is first coated on the Ag substrate through thermal evaporation, and then the Ag nanodisk array is fabricated on the SiO_2_ spacer by some advanced nanofabrication technologies, such as electron beam lithography (EBL).

## Methods

**Fig. 2:**
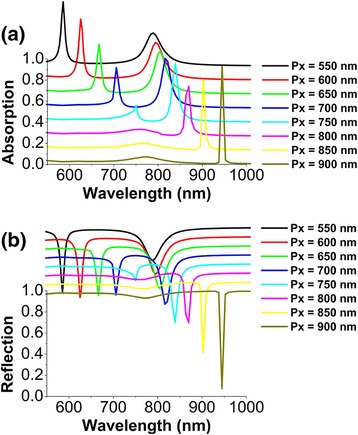
Normal-incidence absorption (**a**) and reflection (**b**) spectra of metamaterials schematically shown in Fig. 1, in the wavelength range from 550 to 1000 nm. The array period *p*_*x*_ along the *x*-axis direction is varied from 550 to 900 nm in steps of 50 nm. The other geometrical parameters: *d* = 150 nm, *h* = 50 nm, *t* = 30 nm, and *p*_*y*_ = 500 nm. For clarity, individual spectra in **a** and **b** are vertically offset by 90 and 60% from one another, respectively

Figure 2 shows the calculated absorption and reflection spectra of a series of metamaterials under normal incidence of light, with the array period *p*_*x*_ along the *x*-axis direction increased from 550 to 900 nm in steps of 50 nm. For each *p*_*x*_, two resonance modes are found in the spectra, which result into the appearance of two absorption peaks and two reflection dips in Fig. 2a and b, respectively. The positions and bandwidths of two resonance modes are strongly dependent on the array period *p*_*x*_. For *p*_*x*_ = 900 nm, the right sharp peak of absorption nearly reaches to 1. Such a strong light absorption in MIM structures is usually called as perfect absorption [64–66]. In addition, we have also investigated the effect of the array period *p*_*y*_ along the *y*-axis direction on the optical properties of metamaterials (not shown here). It is found that simultaneously changing *p*_*y*_ has no significant effect on the optical properties, except for the appearance of a high-order SPP mode when both *p*_*x*_ and *p*_*y*_ are increased to 700 nm. The high-order SPP mode will have an obvious red shift for the array period to be further increased. In Fig. 2, by keeping *p*_*y*_ = 500 nm unchanged, only the lowest order SPP mode propagating in the *x*-axis direction is excited in the spectral range of interest. In the following, we will demonstrate that these two resonance modes originate from the strong coupling between SPPs and MD resonances in the designed metamaterials.

In order to reveal the physical mechanism of two resonance modes in Fig. 2, we have proposed a coupling model of two oscillators to accurately predict the positions of two resonance modes for different array period *p*_*x*_. In the coupling model, one of the oscillators is SPPs, and the other is MD. The strong coupling between SPPs and MD leads to the formation of two hybridized modes, i.e., the high- and low-energy states, whose energies can be calculated by the equation [67]:

$$ {E}_{+,-}=\left({E}_{\mathrm{MD}}+{E}_{\mathrm{SPPs}}\right)/2\pm \sqrt{\Delta /2+{\left({E}_{\mathrm{MD}}-{E}_{\mathrm{SPPs}}\right)}^2/4}. $$

**Fig. 3:**
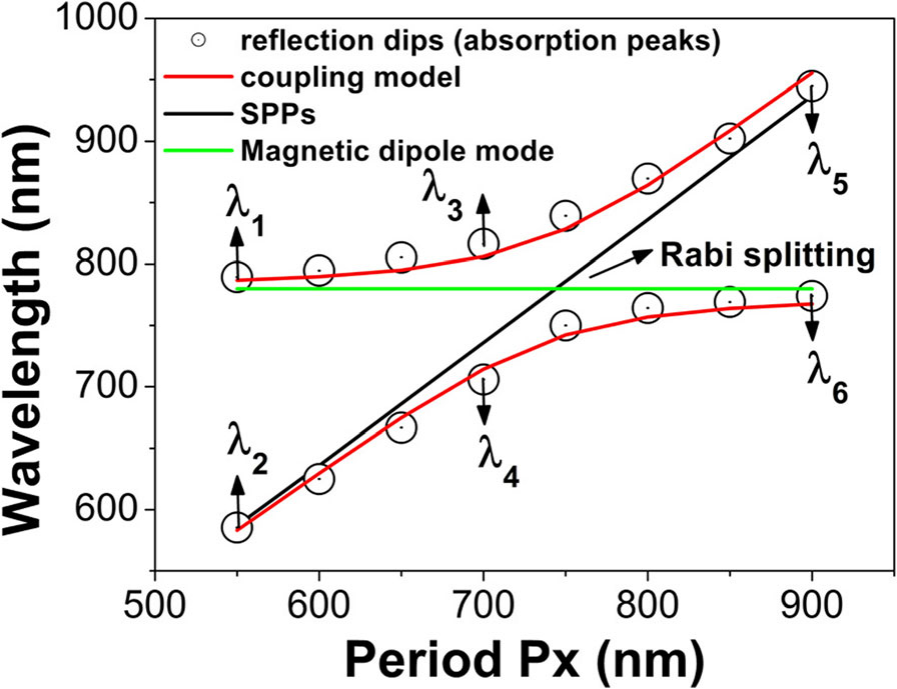
Open black circles show the positions of absorption peaks or reflection dips in Fig. 2, and two red curved lines give the corresponding positions predicted by the coupling model of SPPs and MD mode. The resonance wavelengths of SPPs (black diagonal line) and MD mode (horizontal green line) are also presented

Here, *E*_MD_ and *E*_SPPs_ are the excitation energies of MD and SPPs, respectively; and Δ stands for the coupling strength. In Fig. 3, the open black circles show the positions of two resonance modes for different array period *p*_*x*_, and the two branches of red lines give the corresponding results calculated by the coupled oscillator model with the coupling strength Δ = 100 meV. Obviously, the above model predicted well the positions of two resonance modes. This suggests that the appearance of two resonance modes in Fig. 2 is the result of the interaction of SPPs and MD in metamaterials.

The black diagonal line in Fig. 3 gives the excitation wavelengths of SPPs for different array period *p*_*x*_, which is calculated by matching the reciprocal vector of the Ag nanodisk lattice with the momentum of SPPs under normal incidence [68]. The horizontal green line in Fig. 3 shows the position of MD mode, whose resonance wavelength is mainly determined by the size of Ag nanodisks and the thickness of the SiO_2_ spacer, but is independent of the array periods. At the crossing of the two lines for *p*_*x*_ = 750 nm, SPPs and MD are overlapped in positions, which are strongly coupled together. Therefore, the positions of two resonance modes in Fig. 2 exhibit an obvious anti-crossing, thus forming an interesting phenomenon of Rabi splitting [67]. Far away from the strong coupling regime, the positions of two resonance modes follow approximately one of the two lines.

**Fig. 4:**
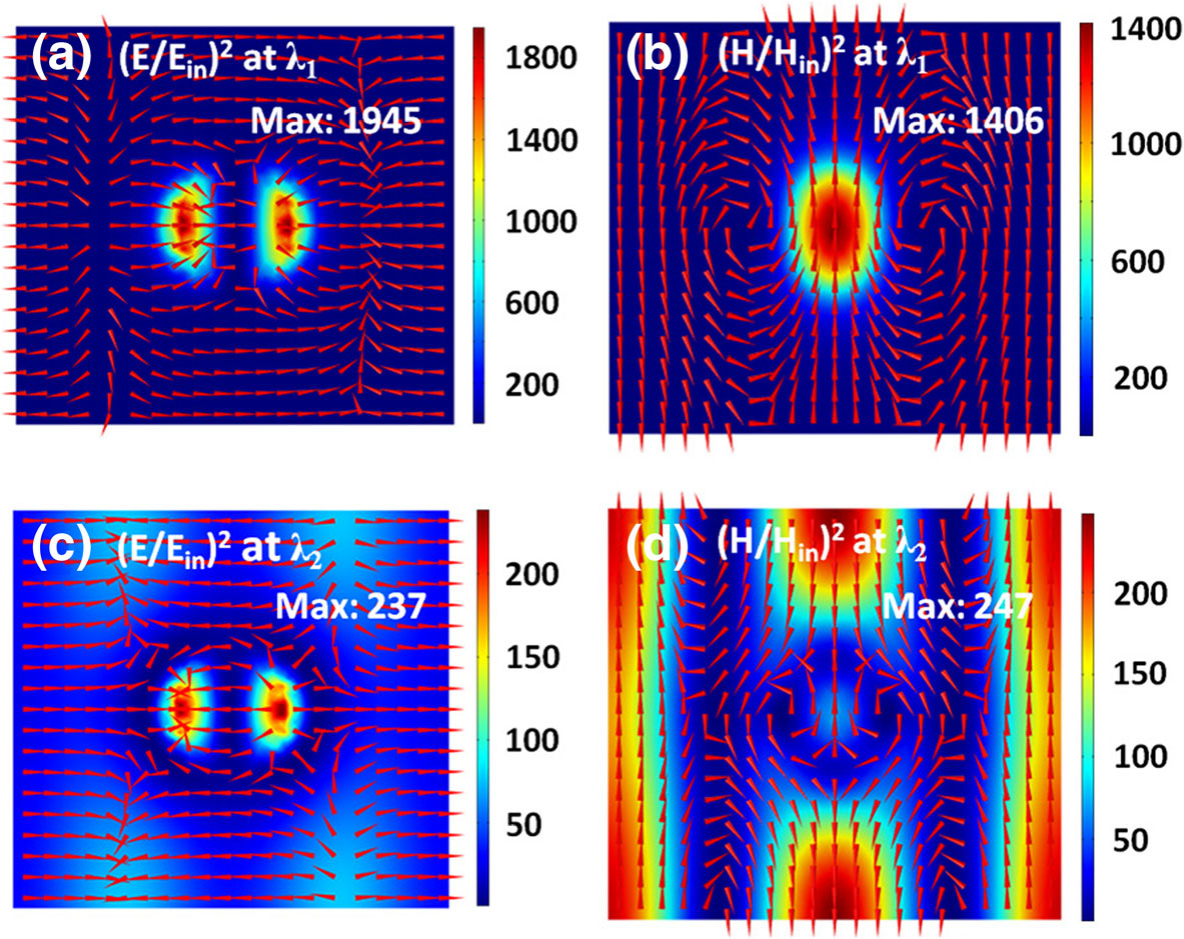
**a**–**d** Normalized electric field intensity (***E***/***E***_in_)^2^ and magnetic field intensity (***H***/***H***_in_)^2^ on the *xoz* plane across the center of the SiO_2_ spacers at the resonance wavelengths of λ_1_ and λ_2_ labeled in Fig. 3. Red arrows represent the field direction, and colors show the field strength

Beside Rabi splitting, another effect of the strong coupling between SPPs and MD is the enhancement of magnetic fields. To exhibit this effect, in Fig. 4, we first plot the distributions of electromagnetic fields at the resonance wavelengths of *λ*_1_ and *λ*_2_ labeled in Fig. 3 for *p*_*x*_ = 550 nm. In this case, the positions of SPPs and MD are far, and their coupling is weak, as exhibited in Fig. 3. At the resonance wavelength of *λ*_1_, the electric fields are highly confined near the edge of the Ag nanodisks and have two field “hotspots” on the left and right sides extending into the SiO_2_ spacer (see Fig. 4a). The magnetic fields are concentrated within the SiO_2_ spacer and have a maximum under the Ag nanodisks (see Fig. 4b). Such distribution properties of electromagnetic fields are mainly the typical characteristics of a MD resonance [69–71]. At the resonance wavelength of *λ*_2_, parallel electromagnetic field bands stretching along the *y*-axis direction are formed, although they are disturbed near the Ag nanodisks (see Fig. 4c and d). In fact, such electromagnetic field distributions mainly correspond to the excitation of SPPs [68].

**Fig. 5:**
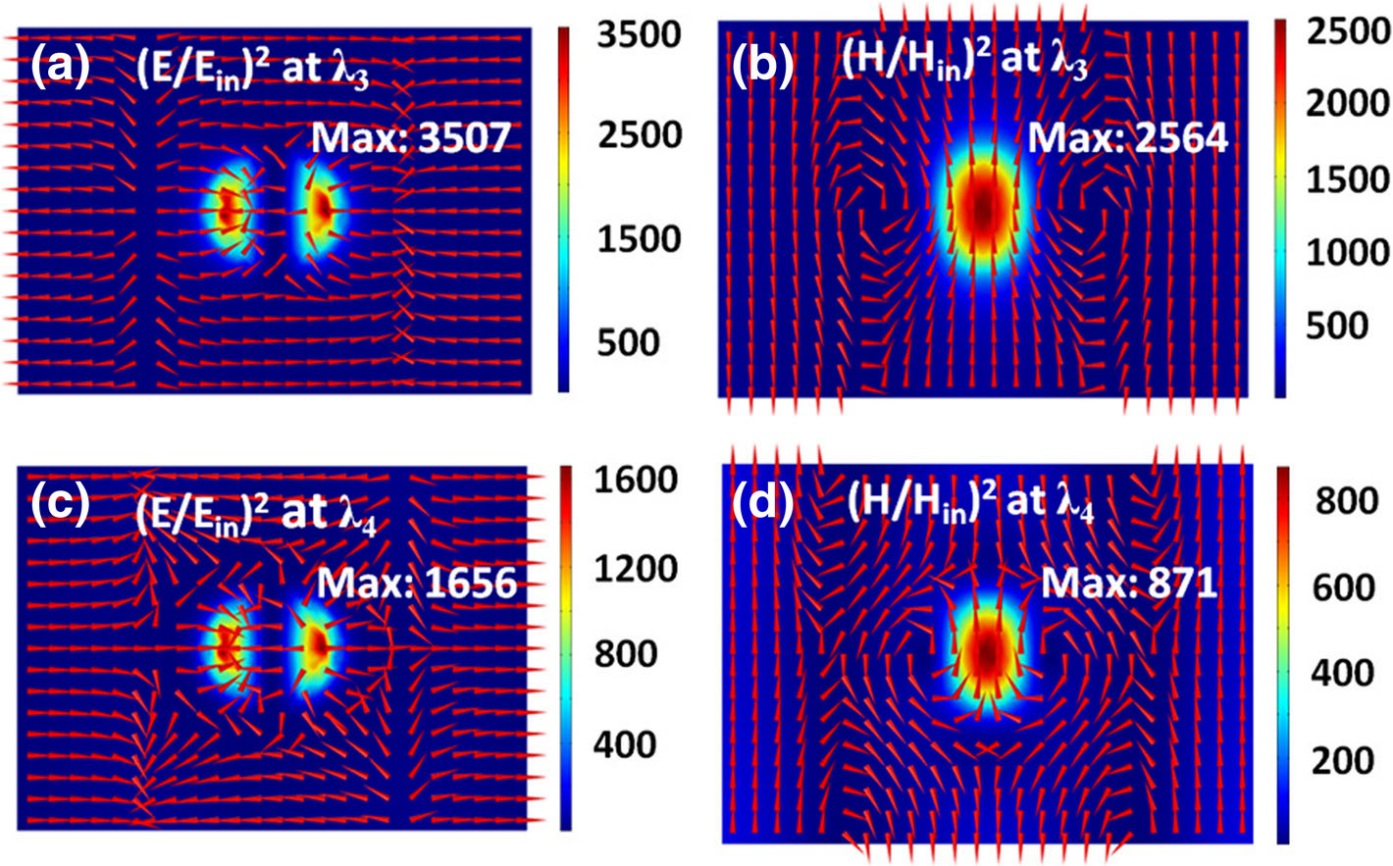
**a**–**d** The same as in Fig. 4 but at the resonance wavelengths of *λ*_*3*_ and *λ*_*4*_ labeled in Fig. 3

In Fig. 5, we plot the distributions of electromagnetic fields at the resonance wavelengths of *λ*_3_ and *λ*_4_ labeled in Fig. 3 for *p*_*x*_ = 700 nm. In this case, the positions of SPPs and MD are close, and their coupling becomes relatively stronger, as exhibited in Fig. 3. As a result, the positions of two resonance modes are red-shifted from *λ*_1_ and *λ*_2_ to *λ*_3_ and *λ*_4_, respectively, and the electromagnetic fields near the Ag nanodisks are further enhanced. As clearly seen in Fig. 5a and b, at the resonance wavelength of *λ*_3_, the maximum electric and magnetic fields are enhanced to be about 3500 and 2560 times of the incident field, which are 1.80 and 1.82 times stronger than the corresponding values at the resonance wavelengths of *λ*_1_, respectively. In Fig. 5c and d, the maximum electric and magnetic fields at the resonance wavelength of *λ*_4_ are enhanced to be about 1650 and 870 times of the incident field, which are 6.98 and 3.53 times stronger than the corresponding values at the resonance wavelengths of *λ*_2_, respectively.

**Fig. 6:**
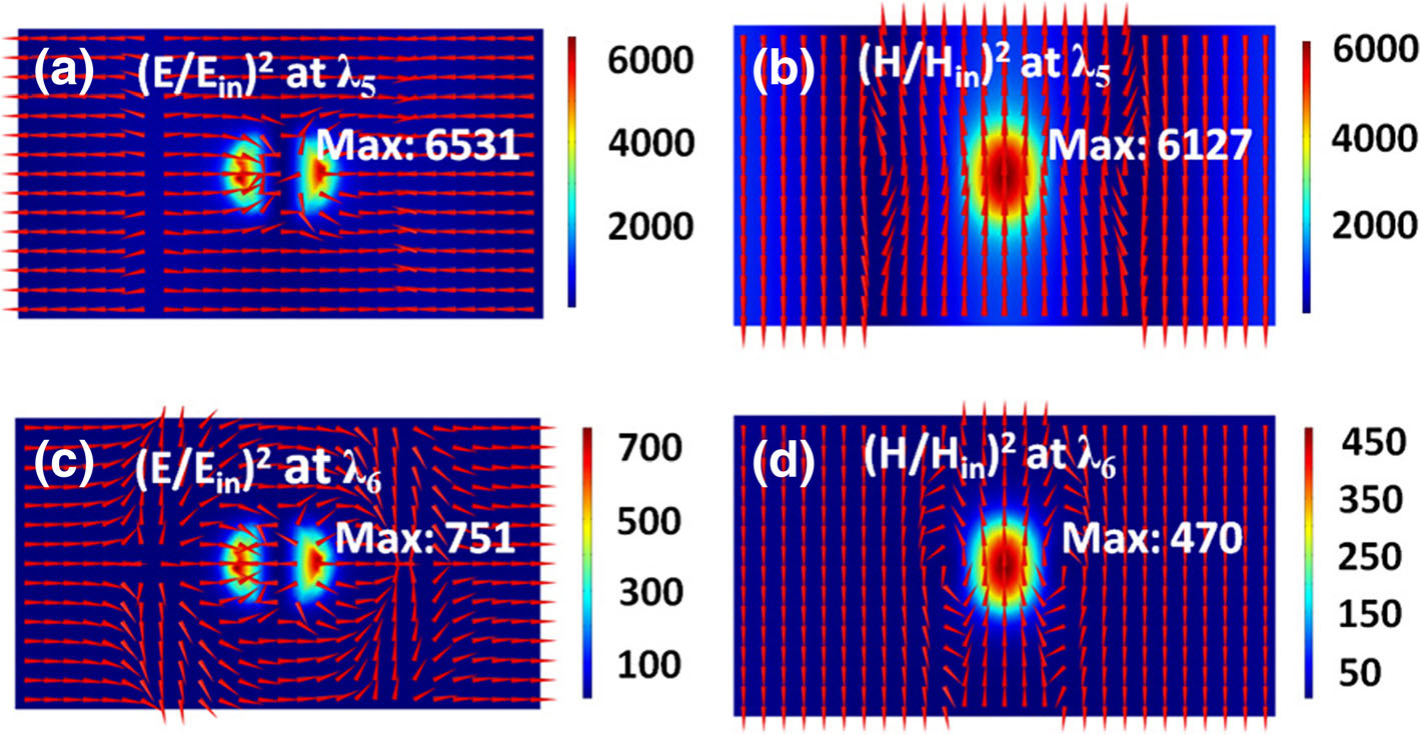
**a**–**d** The same as in Fig. 4 but at the resonance wavelengths of *λ*_*5*_ and *λ*_*6*_ labeled in Fig. 3

Figure 6 shows the electromagnetic field distributions at the resonance wavelengths of *λ*_5_ and *λ*_6_ labeled in Fig. 3 for *p*_*x*_ = 900 nm. The mixed mode at *λ*_5_ has a very narrow bandwidth, as clearly seen in Fig. 2. As a result, its electromagnetic fields are hugely enhanced, with the maximum electric and magnetic fields exceeding 6500 and 6100 times of the incident fields, respectively. The huge enhancement of electromagnetic fields may find potential applications in nonlinear optics and sensing [72, 73]. In Fig. 6b, there exist three relatively weak field enhancement bands parallel in the *y*-axis direction and a pronounced field hotspot at the center. Such a field distribution directly indicates the hybridization feature of SPPs and MD. The mixed mode at *λ*_6_ has a broad bandwidth, which has more component of MD than SPP, as indicated in Fig. 6c and d.

## Conclusions

In this work, we have numerically investigated the coupling effects of SPPs and MD resonances in metamaterials, which are composed of an Ag nanodisk array and a SiO_2_ spacer on an Ag substrate. The near-field plasmon interactions between individual Ag nanodisks and the Ag substrate form MD resonances. The periodicity of the Ag nanodisk array leads to the excitation of SPPs at the surface of the Ag substrate. When the excitation wavelengths of SPPs are tuned to be close to the position of MD resonances by varying the array period of Ag nanodisks, SPPs and MD resonances are coupled together into two hybridized modes, whose positions can be accurately predicted by a coupling model of two oscillators. In the strong coupling regime of SPPs and MD resonances, the hybridized modes exhibit an obvious anti-crossing and, thus, result into an interesting phenomenon of Rabi splitting. At the same time, the magnetic fields under the Ag nanodisks are enhanced greatly, which may find some potential applications, such as magnetic nonlinearity.
